# Pharmacogenomics of Impaired Tyrosine Kinase Inhibitor Response: Lessons Learned From Chronic Myelogenous Leukemia

**DOI:** 10.3389/fphar.2021.696960

**Published:** 2021-06-28

**Authors:** Meike Kaehler, Ingolf Cascorbi

**Affiliations:** Institute of Experimental and Clinical Pharmacology, University Hospital Schleswig-Holstein, Kiel, Germany

**Keywords:** drug resistance, CML, BCR-ABL, CYP3A4, ABCB1, ABCG2, OCT1, miRNA

## Abstract

The use of small molecules became one key cornerstone of targeted anti-cancer therapy. Among them, tyrosine kinase inhibitors (TKIs) are especially important, as they were the first molecules to proof the concept of targeted anti-cancer treatment. Since 2001, TKIs can be successfully used to treat chronic myelogenous leukemia (CML). CML is a hematologic neoplasm, predominantly caused by reciprocal translocation t(9;22)(q34;q11) leading to formation of the so-called BCR-ABL1 fusion gene. By binding to the BCR-ABL1 kinase and inhibition of downstream target phosphorylation, TKIs, such as imatinib or nilotinib, can be used as single agents to treat CML patients resulting in 80 % 10-year survival rates. However, treatment failure can be observed in 20-25 % of CML patients occurring either dependent or independent from the BCR-ABL1 kinase. Here, we review approved TKIs that are indicated for the treatment of CML, their side effects and limitations. We point out mechanisms of TKI resistance focusing either on BCR-ABL1-dependent mechanisms by summarizing the clinically observed BCR-ABL1-mutations and their implications on TKI binding, as well as on BCR-ABL1-independent mechanisms of resistances. For the latter, we discuss potential mechanisms, among them cytochrome P450 implications, drug efflux transporter variants and expression, microRNA deregulation, as well as the role of alternative signaling pathways. Further, we give insights on how TKI resistance could be analyzed and what could be learned from studying TKI resistance in CML *in vitro*.

## Introduction

The development of tyrosine kinase inhibitors tremendously changed anti-cancer drug therapy and opened new treatment options and strategies. Successfully enabling new therapy regimen by specific blockade of the ATP-binding domain of a tyrosine kinase led to increased patient survival rates, less side effects and improved outcome for the patients. Initially established for the use in chronic myelogenous leukemia (CML) by targeting the BCR-ABL1 fusion protein, the outstanding therapeutic success made tyrosine kinase inhibitors a prominent example of the concept of targeted therapy. Meanwhile, there are multiple therapeutic options in which tyrosine kinase inhibitors (TKIs) are first-line choice in therapy or co-therapy, i.e. targeting epidermal growth factor receptor (EGFR) subtypes using erlotinib or gefitinib in HER1-overexpressing tumors, as well as lapatinib to inhibit HER2 in HER2-positive breast cancer, targeting angiogenesis *via* vascular endothelial growth factor receptor VEGF(R) inhibition or blockade of kinases, such as c-kit (CD117), platelet derived growth factor receptor (PDGFR), or anaplastic lymphoma kinase (ALK), just to name a few (Jiao et al., 2018). Nevertheless, acquired therapy resistances occur during the treatment with TKIs. Here, we review the TKIs used in CML regarding their side effects and limitations. Moreover, we discuss potential mechanisms of impaired TKI response in CML, in particular genomics of *BCR-ABL1*, the impact of variants in cytochrome P450 enzymes and drug transporters, as well as alternative mechanisms of resistance. In addition, we summarize what can be learned from CML for the treatment other neoplasms.

### Role Model of Successful TKI-Based Anti-cancer Therapy: Chronic Myelogenous Leukemia

The hematopoietic neoplasm chronic myelogenous leukemia (CML) is a rare disorder predominantly caused by reciprocal translocation t (9; 22) (q34; q11) resulting in formation of the so-called Philadelphia chromosome (Ph) and the *BCR-ABL1* fusion gene (Nowell and Hungerford, 1960; Rowley, 1973; Heisterkamp et al., 1983). This fusion gene makes up for 95% of all CML and 20% of Ph + acute lymphatic leukemia (ALL) cases and is the main driver of malignant cell progression in these leukemias (Radich, 2001; Soverini et al., 2019). For several decades, CML has been a fatal disease with hardly any effective treatment using arsenic substances, radiotherapy, cytostatic drugs, i.e., busulfan and hydroxyurea, or interferon-α, with the latter compounds at least resulting in normalization of the blood visible as hematological remission or even cytogenetic response (Kennedy, 1972; Morstyn et al., 1981; Hukku et al., 1983; Talpaz et al., 1987). Nevertheless, since the development of a tyrosine kinase inhibitor targeting BCR-ABL1 in the 90s century, CML can be effectively treated using the 2-phenyl-aminopyrimidine imatinib resulting in more than 80% 10-years survival rates in a life-long treatment regimen (Druker et al., 1996; Hochhaus et al., 2017). Since then, tyrosine kinase inhibitors, in particular imatinib, became first-line therapy in CML superseding previous treatment strategies (Hochhaus et al., 2020). This showed for the first time that kinases can be used as druggable targets for anti-cancer treatment. Nevertheless, CML requires a life-long treatment with the respective TKI, as discontinuation might provoke relapses of remaining CML cells. Although several markers are considered to identify suitable patients for therapy termination, e.g. duration of therapy or response rate before discontinuation, *BCR-ABL1/ABL1* ratio, or Sokal score, median relapse rate of patients is approximately 51% (Campiotti et al., 2017; Etienne et al., 2017). Therefore, further studies are needed to identify eligible patients to safely discontinue the treatment.

### Tyrosine Kinase Inhibitors in CML: Indications, Side Effects and Treatment Limitations

The fusion gene *BCR-ABL1* arises from the breakpoint cluster region (*BCR*) and the Abelson tyrosine kinase 1 (*ABL1*). While the physiological function of the phosphoprotein BCR is relatively unclear, ABL1 encodes for a cytosolic tyrosine kinase involved in the regulation of proliferation (McCubrey et al., 2008; Bixby and Talpaz, 2011). In *Ph +* cells, BCR-ABL1 is constitutively active, which results in malignant progression. Imatinib binds to the type II conformation of BCR-ABL1 and inhibits binding of ATP to the ATP binding domain preventing phosphorylation of downstream target proteins (Druker et al., 1996; Nagar et al., 2002). This results in proliferation stop and apoptotic cell death. Besides, BCR-ABL1, imatinib also binds to other tyrosine kinases: ABL1 and ABL2 (also named Abelson-related gene ARG), the membrane kinase c-kit (CD117), platelet-derived growth factor receptor beta (PDGFRβ) and colony stimulating factor 1 (M-CSF) (Buchdunger et al., 1995; Buchdunger et al., 1996; Heinrich et al., 2000; Dewar et al., 2005). While inhibition of both ABL paralogs might contribute to the observed side effects of imatinib treatment (Buchdunger et al., 1996), imatinib is used to target c-kit-mutated gastrointestinal stroma tumors (GIST) or PDGFRβ-mutated chronic myelomonocytic leukemia (CMML, Table 1) (Poveda et al., 2017; Valent et al., 2019). For inhibition of M-CSF, the influence on therapeutic outcome or side effects remains unclear (Dewar et al., 2005). The occurring side effects of imatinib treatment (but also of later generation TKIs) are gastrointestinal disorders, i.e. nausea or emesis, dermatitis, and in severe cases leukocytopenia, heart failure or liver disorders (Hahn et al., 2003; Kalmanti et al., 2015; Steegmann et al., 2016). Although the side effects are much less severe compared to classical chemotherapy using cytostatic drugs and no absolute contraindications or life-threatening complications have been observed yet, in approximately 10% of patients, distinctive side effects lead to interruption or termination of the therapy with the majority occurring over time or after a drug holiday (O'Brien et al., 2003a; Hochhaus et al., 2020).

**TABLE 1:** Therapeutic targets, impact of metabolic pathways and drug transporters of tyrosine kinase inhibitors, used for the treatment of CML.

Tyrosine kinase inhibitor	Therapeutic target	CYP3A4/5	OCT1	ABCB1	ABCG2
Imatinib	BCR-ABL1 PDGFRβ c-KIT	+	?	+	+
Nilotinib	BCR-ABL1 PDGFRβ c-KIT CSF-1R DDR	+	?	+	+
Dasatinib	Multi kinase inhibitor BCR-ABL1 src family PDGFRβ c-KIT	+	?	+	+
Bosutinib	Dual BCR-ABL1/Src inhibitor	+	?	−	-
Ponatinib	BCR-ABL1 T315I	+	?	+	+

Adapted from Deng et al., 2014. +: strong evidence, substrate or inhibitor; -: no evidence; ?: evidence unclear.

While the use of tyrosine kinase inhibitors in CML is tremendously successful, approximately 20–25% of all treated CML patients suffer from loss of previously achieved cytogenetic or major molecular response within 5 years of treatment (Milojkovic and Apperley, 2009; Hochhaus et al., 2017). This stresses the utter need for treatment alternatives. For this purpose, the second and third generation TKIs were developed. Besides imatinib, there are four clinically approved tyrosine kinase inhibitors namely second-generation inhibitors nilotinib, dasatinib and bosutinib and third-generation ponatinib, which differ in their potency, side effects, targets and efficacy against BCR-ABL mutations. Nilotinib, which also binds to the inactive conformation of BCR-ABL1, is 20-fold more potent than imatinib, but also binds to mitogen activated protein (MAP)-kinases and might provoke cardiovascular events in 20% of patients more frequently than imatinib (5%) (Manley et al., 2010; Hughes et al., 2019a). In addition, cerebrovascular events, hypertension, hypercholesterolemia, diabetes as well as pancreatitis are contradictory (Rosti et al., 2009). Besides similar adverse effects compared to imatinib, the second generation TKI dasatinib, which binds to the active BCR-ABL1 conformation, is likely to cause pleuro-pulmonary toxicity or pleural effusion in approximately 37% of the patients, while being less specific (Kitagawa et al., 2013; Cortes et al., 2016). The broad specificity SRC/ABL inhibitor bosutinib, which was initially designed to inhibit SRC in SRC-overexpressing tumors, but also shows high activity against ABL (and BCR-ABL) (Keller et al., 2009), binds to the BCR-ABL1 kinase independent from the kinase conformation, while provoking transient diarrhea in about 30% of patients (Remsing Rix et al., 2009). In addition, increased levels of transaminases might be a temporary side effect (Hochhaus et al., 2020).

Ponatinib is considered to be a second line TKI used in case of T315I mutation (see below) and resistance to first or second generation TKIs (Cortes et al., 2013). Compared to the other TKIs, the highest number of adverse events occurs during treatment with 30% cardiovascular toxicity and cardiovascular risk factors being contraindicated. Further, the risk of arterial occlusion events should be considered by monitoring hypertension, hyperlipidemia, diabetes and smoking cessation (Hochhaus et al., 2020). Ponatinib binds to the inactive state, precisely the DFG (Asp-Phe-Gly)-out motif, of BCR-ABL1. It should be added that treatment with TKIs is especially effective in chronic phase CML, while the treatment of advanced phases or terminal blast crises, which became rare due to excellent response rates, includes classical chemotherapy or allogenic stem cell transplantation (comprehensively summarized in (Hochhaus et al., 2020).

Regarding genomics of adverse events, little is known about the relevance of SNVs during TKI treatment of CML. Overall, it seems that drug-drug interactions or variants in drug transporters play a more important role in drug resistance than in the occurrence of adverse events (see below).

### Genomics of Therapy Resistances: *BCR-ABL1*-Mutations

About approximately 50% of all TKI resistances in CML occur due to mutations or overexpression/amplification of the BCR-ABL1 kinase leading to loss of TKI binding and re-activation of the downstream phosphorylation cascade (Gorre et al., 2001; Jabbour et al., 2011; Baccarani et al., 2013; Rosti et al., 2017). BCR-ABL1 consists of the breakpoint cluster region protein and the tyrosine kinase ABL. The latter is structured by the *N*-terminal lobe and *C*-terminal lobe fused by a hinge region. In the *N*-lobe, *ß*-sheets and an *a*-helix, as well as an SRC-homology domain regulating the tyrosine kinase activity are located. The two *ß*-sheets are fused by a P-loop, which contributes to binding of ATP. In the *C*-lobe, the ATP binding site and the activation loop with conserved DFG required for kinase activation (aspartate, phenylalanine, glycine 381–383) are situated (Reddy and Aggarwal, 2012). Imatinib binds to the inactive conformation of the BCR-ABL1 ATP binding pocket and requires six hydrogen bonds and the conformation switch of activation domain and P-loop into the active conformation (Reddy and Aggarwal, 2012). Therefore, mutations altering the necessary amino acids can tremendously limit the function of the drug (Eiring and Deininger, 2014). Binding of imatinib is entirely abolished by the so-called gatekeeper mutation T315I, in which one hydrogen bond is removed inside the ATP binding pocket. This mutation also leads to loss of action of the second generation TKIs. The only remaining treatment option to this date is ponatinib, which is a pan-BCR-ABL1 inhibitor and binds to the ATP binding domain independent from the T315 hydrogen bond, although this mutation requires increase of the ponatinib dose (O'Hare et al., 2012; de Lavallade and Kizilors, 2016; Braun et al., 2020; Luciano et al., 2020). Nevertheless, a second step mutation on the same residue from isoleucine to methionine results in failure of ponatinib as well (Zabriskie et al., 2014). Besides these TKIs, the allosteric inhibitor of ABL1 asciminib, as a mimic of the *N*-terminal myristoyl group of ABL1 (and therefore named specifically targeting the ABL myristoyl pocket-(STAMP)-inhibitor), might be an alternative to overcome resistances due to BCR-ABL1 mutations, which are located in the ATP binding domain. As the myristoyl group is lost in the BCR-ABL1 fusion protein, autoregulation of ABL1 is prevented resulting in malignant activation of the signaling transduction cascade, which might be overcome by asciminib (Schoepfer et al., 2018; Hughes et al., 2019b; Eide et al., 2019).

**FIGURE 1:**
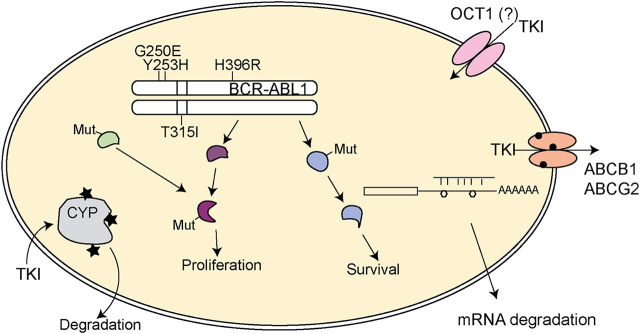
Schematic representation of pharmacogenetic variants leading to TKI resistance in CML. Mutations in BCR-ABL1 (depicted by the protein loci of the mutation) can lead to TKI loss of function. Further, mutations in downstream signaling pathways (Mut) might provoke constitutive activation of the pathway or may lead to activation of alternative signaling pathways that undertake the signaling transduction to sustain proliferation and survival of the tumor cell. Variants in cytochrome 450 enzymes (indicated by stars) could facilitate loss of metabolism of the respective TKI and thereby impaired turnover. In addition, variants in ABCB1 or ABCB2 (circles) might lead to altered TKI efflux and TKI response. The general role of the drug importer OCT1 is still controversially discussed. SNVs in mRNAs (polygons) can also lead to impaired binding of microRNAs, which itself results in altered gene expression potentially contributing to TKI resistance. TKI: tyrosine kinase inhibitor.

Moreover, mutations in the P-loop, i.e. G250E or Y253H, destabilizing binding of imatinib or in the activation loop, i.e. H396R, prevent the activation loop to maintain the closed position lead to imatinib failure (Reddy and Aggarwal, 2012). However, nilotinib is known to fail as well in the two depicted mutations in the P-loop, while bosutinib is partially resistant to G250E, but a therapeutic option in Y253H (Soverini et al., 2014). This shows the utter need for stratification by the BCR-ABL1 mutation pattern to determine to best TKI for the therapy (Table 2; Figure 1). This is also the case for patients with intolerance to one distinct TKIs.

**TABLE 2:** Examples of mutations in the BCR-ABL1 protein and their influence on TKI response in CML.

Protein mutation	Localisation in the protein	Consequence on structure, TKI binding	Clinical options
**BCR-ABL1**			
T315I	ATP binding pocket	Loss of binding of imatinib, nilotinib, dasatinib, bosutinib	Switch to ponatinib
T315M	ATP binding pocket	Second step mutation, loss of function of ponatinib	No treatment option, asciminib?
G250E	P-loop	Failure of imatinib, nilotinib, bosutinib	Use of dasatinib or ponatinib
Y253H	P-loop	Failure of imatinib, nilotinib	Switch to dasatinib, bosutinib or ponatinib
H396R	Activation loop	Failure of imatinib	Switch to second generation TKIs

Adapted from Hochhaus et al., 2020; de Lavallade et al., 2016; Soverini et al., 2014; Zabriskie et al., 2014.

### Genomics of Therapy Resistances: BCR-ABL1-independent Mechanisms

Besides mutations in BCR-ABL1, resistances can occur independently from the kinase. These include multiple aspects, which will be reviewed thereafter (Figure 1).

### Impact of Drug Metabolism: *CYP3A4/CYP3A5*

TKIs are substrates for cytochrome P450, mainly for CYP3A4 and CYP3A5 (Haouala et al., 2011). Therefore, it is not surprising that drug-drug interactions may occur with a large number of co-medications causing induction or inhibition of this metabolic pathway. These include rifampicin, anticonvulsants, i.e. carbamazepine, or herbal products, e.g. St. John’s wort, that are confirmed PXR ligands inducing certain cytochrome P450 enzymes including CYP3A4 and 3A5. As a consequence, enhanced metabolism of TKIs diminishes the striven TKI plasma concentration contributing to chemoresistance (Peng et al., 2005; Tian et al., 2018). Imatinib itself is considered to be a moderate CYP3A4 inhibitor, while being a substrate (O'Brien et al., 2003b; Filppula et al., 2012). CYP3A4 metabolizes imatinib to the active, but less cytotoxic metabolite N-desmethyl-imatinib (CPG74588) (Mlejnek et al., 2011). Interestingly, the autoinhibition of CYP3A4 reveals a second pathway, namely CYP2C8, to be involved in hepatic elimination after imatinib exposure (Filppula et al., 2013). Moreover, it was observed that a higher activity of CYP3A4 and CYP3A5 was present in CML patients achieving complete molecular remission compared to poor responders (Green et al., 2010). It was discussed whether pharmacological long-acting metabolites would have contributed to this observation.

Similar to the role of the enzyme activity, the presence of pharmacogenetic variants might limit enzyme activity and thereby affecting the metabolism of imatinib. The main clinically relevant polymorphisms are CYP3A4*20 (rs67666821) expressed as a truncated protein with loss in enzymatic activity and CYP3A4*22 (rs35599367) resulting in loss of about 20% enzyme activity, while evidence for variants with increased enzyme activity is lacking (Werk and Cascorbi, 2014; Saiz-Rodriguez et al., 2020). *CYP3A4* and *CYP3A5* share a high sequence homology and overlap in their substrate spectra (Williams et al., 2002). For *CYP3A5*, the main variants are non-functional CYP3A5*3 (rs776746), CYP3A5*6 (rs10264272), CYP3A5*7 (rs41303343) that differ in their expression patterns between the ethnicities (Kuehl et al., 2001; Werk and Cascorbi, 2014). CML patients with known *CYP3A4* polymorphisms might suffer from impaired TKI metabolism resulting in increased adverse effects, but presumably also the response to the TKI might be improved. However, there is conflicting data on the role of *CYP450* variants on the response to TKIs. Interestingly, for CYP3A5*3, inferior imatinib response of the variant compared to wild-type carriers was observed in several studies contradicting the presumption of improved imatinib response in the presence of a non-functional CYP3A5 protein (Liu et al., 2002; Kim et al., 2009; Bedewy and El-Maghraby, 2013; Harivenkatesh et al., 2017). In contrast, a meta-analysis for CYP3A5*3 revealed an association of higher complete cytogenetic response rates under imatinib treatment at least in the Asian population (Cargnin et al., 2018). However, future conformational studies are necessary to confirm these findings in other cohorts. An association of the TKI response to the other variants mentioned has not been fully elucidated, yet some studies point to a contribution of CYP2C8*2 and CYP3A4*7 to alterations in imatinib trough levels in homozygous carriers resulting either in an increase or decrease in the concentration (Adehin et al., 2019). The main genetic variants are summarized in Table 3. Regarding adverse events, it seems that CYP3A4 interactions play a larger role in adverse events or lack of TKI response than genetic variants in CYP3A4, as observed for e.g. phenytoin, cyclosporin A or ketoconazole (Dutreix et al., 2004; Atiq et al., 2016; Osorio et al., 2019). Therefore, assessment of cytochrome P450 genotypes or function is not performed in the clinical routine to this date. Further studies are necessary to analyze the relevance of these enzymes in relation to drug resistance and adverse events.

**TABLE 3:** Pharmacogenetic variants in cytochrome P450 enzymes and in drug transporters and their relevance to TKI response in CML.

Pharmacogenetic variant	Rs-number	Consequence	Evidence
**Cytochrome P450 enzymes**			
CYP3A4*20	rs67666821	Truncated protein	—
CYP3A4*22	rs35599367	Intronic SNP, C > T	—
CYP3A5*3	rs776746	Cryptic splice site with premature stop codon, A > G	Unclear, contradictory data
CYP3A5*6	rs10264272	Synonymous, G > A	—
CYP3A5*7	rs41303343	Insertion, frameshift mutation	Decrease in imatinib trough concentration?
CYP2C8*2	rs11572103	Missense, T > A	Increase in imatinib trough level?
**Drug transporters*OCT1***			
181C > T	rs1208357	R61C	—
480C > G	rs683369	L160F	—
1022C > T	rs2282143	P341L	—
1222A > G	rs628031	M408V	—
1260-1262delGAT	rs72552763	M420del	—
***ABCB1***			
1199G > A/T	rs2229109	S400 N/L	Relevance unclear
1236C > T	rs1128503	Synonymous	Increased imatinib response?, no association to nilotinib, dasatinib, ponatinib
2677G > T/A	rs2032582	A893 S/T	Increased imatinib response?, no association to nilotinib, dasatinib, ponatinib
3435C > T	rs1045642	Synonymous	Increased imatinib response?, no association to nilotinib, dasatinib, ponatinib
***ABCG2***			
34G > A	rs2231137	V12M	Improved response to imatinib?
421C > A	rs2231142	Q141K	Conflicting data
−15,622C > T	rs7699188	Low expression of BCRP	Unclear?

Adapted from Werk and Cascorbi, 2014; White et al., 2006; Watkins et al., 2015; Bruckmueller and Cascorbi, 2021; -: lack of evidence.

### Impact of Drug Transporters

Besides hepatic metabolism, drug transporters are known to be involved in drug resistance impairing the intracellular drug concentration or limiting the bioavailability of a drug in certain tissues. For CML, several drug transporters are discussed being either drug importers or efflux transporters (see Table 3).

### OCT1

The organic cation transporter 1 *OCT1/SLC22A1* is considered to be involved in the import of some TKIs into the tumor cells. However, data regarding its relevance in CML is controversial, as an upregulation of *OCT1* in imatinib resistance was shown (White et al., 2006; Engler et al., 2010), while others clearly demonstrated the absence of a *OCT1* regulation (Davies et al., 2009; Nies et al., 2014). Interestingly, it was shown that OCT1 expression and activity might be used as a prognostic marker for long-term imatinib response of CML patients (Watkins et al., 2015). Regarding pharmacogenetics, the main variants in *OCT1* are 181C > T (R61C, rs12208357), 480C > G (L160F, rs683369), both located in exon 1; exon 6 1022C > T (P341L, rs2282143), 1222A > G (M408V, rs628031) and 1260-1262delGAT (M420del, rs72552763), both located in exon 7. Nevertheless, several studies did not confirm an influence of any *OCT1* variant on imatinib response (Watkins et al., 2015).

### ABC Transporters

Regarding drug efflux transporters, the CML TKIs are discussed to be dose-dependent substrates or inhibitors of P-glycoprotein (P-gp, ABCB1) and breast cancer resistance protein (BCRP, ABCG2) being drug efflux transporters of the ATP binding cassette (ABC) family that limit the intracellular concentration of the respective TKI (Hegedus et al., 2009; Anreddy et al., 2014; Beretta et al., 2017). In particular imatinib, nilotinib, dasatinib and ponatinib were shown to be substrates of both, ABCB1 and ABCG2, whereas bosutinib shows only little affinity and cannot be considered as substrate of one of the mentioned ABC transporters (Deng et al., 2014). Being overexpressed, these transporters are known to contribute to drug resistance in several tumors (Li et al., 2016; Mohammad et al., 2018). Besides questions on drug competition and varying expression of these ABC transporters, pharmacogenetic variants in *ABCB1* or *ABCG2* might have an impact on the development of drug resistance (Bruhn and Cascorbi, 2014; Kaehler and Cascorbi, 2019).

### ABCB1

*ABCB1* is one of the most extensively investigated drug transporters and broadly analyzed in terms of pharmacogenetic variants. It could be expected that loss of function variants or those with impaired protein function resulting in reduction in efflux capability may lead to improved response to TKIs. However, so far there is no clear evidence that *ABCB1* variants could be applied as predictive biomarkers in any drug therapy (Bruckmueller and Cascorbi, 2021). Most pharmacogenetic studies on TKIs focused on the common variants are 1236C > T (synonymous, exon 12, rs1128503), 2677G > T/A (A893 S/T, exon 21, rs2032582) and 3435C > T (synonymous, exon 26, rs1045642). Regarding response to imatinib, there is conflicting data. Whereas in vitro-experiments using *ABCB1*-overexpressing cells demonstrated a moderately increased imatinib response in triple variants carriers compared to wild-type (Dessilly et al., 2016b), a comprehensive meta-analysis of clinical studies revealed lack of significance on molecular response in relation to any of the above mentioned *ABCB1* variants (Wang et al., 2015). In addition, the role of these variants during treatment with nilotinib, dasatinib and ponatinib also lacked a clear association (Dessilly et al., 2016b; Galimberti et al., 2017). Regarding less common variants, the influence of 1199G > A/T is also controversially discussed, as for the A variant allele increased efflux of imatinib, nilotinib and dasatinib was observed *in vitro*, while this finding was not detected in other studies (Skoglund et al., 2013; Dessilly et al., 2016a). Overall, the role of *ABCB1* polymorphisms in TKI resistance remains controversial. At least, variants do not seem to be suitable as predictive biomarkers of drug response.

### ABCG2

Besides its function as drug efflux transporter, ABCG2 is also regarded as stem cell factor being highly expressed in hematopoietic precursor and stem cells (Scharenberg et al., 2002; Jordanides et al., 2006). Similar to *ABCB1*, *ABCG2* polymorphisms are discussed to alter the transport capability of this protein. The most important variants are 34G > A (V12M, exon 2, rs2231137) and 421C > A (Q141K, rs2231142). Some evidence pointed to homozygous 34G > A resulting in amino acid exchange from valine to methionine to be associated with an improved response to imatinib potentially due to reduction in *ABCG2* expression (Kim et al., 2009). For 421C > A, which presumably affects the conformation of the ATP binding domain, data is conflicting as it was shown that expression of the variant limited imatinib bioavailability, while others demonstrated no effects on the pharmacokinetics of imatinib *in vivo* (Gardner et al., 2006; Takahashi et al., 2010; Skoglund et al., 2014). Nevertheless, Jiang and colleagues suggested a potential use of this variant to predict imatinib response in CML (Jiang et al., 2017). In addition to these polymorphisms, the -15,622C > T promoter SNP (rs7699188) was associated with low expression of BCRP in multiple tissues, including the liver, likely to decrease imatinib clearance from the cell (Poonkuzhali et al., 2008). Additional variants in *ABCG2* were also analyzed, but revealed hardly any effects on TKI clearance or response (Bruckmueller and Cascorbi, 2021). To conclude, for both, ABCB1 and ABCG2, a clear association of pharmacogenetic variants to imatinib response is lacking and future studies are necessary to provide insights into their relevance in drug resistance.

Adding to the complexity, expression of ABCB1 and ABCG2 in drug resistance seems to be dose-dependent, as in several studies controversial findings were observed pointing to a dynamic expression of these proteins (Gromicho et al., 2011; Eadie et al., 2013; Kaehler et al., 2017). Interestingly, it was shown that ABCG2 expression in peripheral blood leukocytes could be used to predict treatment-free remission during imatinib discontinuation (Rinaldetti et al., 2018). Nevertheless, future studies are needed to analyze the influence of ABC transporter variants in neoplasms, such as CML.

### Epigenetics and microRNAs

Besides activation or repression by transcription factors, gene expression is regulated by epigenetic factors. These imply DNA methylation or histone modifications as acetylation or ubiquitinoylation, as well as post-transcriptional regulation. For CML, there is some evidence on the influence of methylation during the progression of the CML phases, as it was shown that the *ABL1* promoter is hypermethylated in early stages of CML, as well as a global hypermethylation in CML blast crisis occurs (Machova Polakova et al., 2013; Heller et al., 2016). In TKI drug resistance, an increase in overall methylation was also observed in patients resistant or intolerant to imatinib (Jelinek et al., 2011). However, these findings are limited on distinct genes.

Besides epigenetic regulation, expression of microRNAs might be involved in the pathogenesis of CML and drug resistance. microRNAs are 19–21 nt short ribonucleotides involved in post-transcriptional regulation of gene expression by binding specifically to the 3’ UTR of their target mRNAs and provoking either their degradation or translational stop (Kim, 2005; Krutzfeldt et al., 2006). As microRNAs regulate expression of tumor suppressor or oncogenes, aberrant microRNA-expression was demonstrated in several malignancies, as well as in combination with anti-cancer drugs (Zheng et al., 2010). In CML, it was shown that the presence of TKIs alters the microRNA expression pattern in blood samples of CML patients (Flamant et al., 2010). In addition, the global microRNA expression pattern seems to differ between either drug sensitivity and resistance *in vitro*, in CML patients, as well as in responding and non-responding CML patients or CML phases (San Jose-Eneriz et al., 2009; Machova Polakova et al., 2011; Turrini et al., 2012; Klumper et al., 2020). Moreover, distinct microRNAs, as shown e.g. for miR-203 or -30a/e, target the *BCR-ABL1* gene and their deregulation might contribute to altered response to TKIs (Liu et al., 2013; Shibuta et al., 2013; Hershkovitz-Rokah et al., 2014). Even beyond BCR-ABL1, fine-tuning of gene expression by microRNAs as e.g. *MYC* by miR-144/451 or miR-212/*ABCG2* might be involved to regulate the relevant target genes in the downstream signaling cascade and contribute to drug resistance (Liu et al., 2012; Kaehler et al., 2017). Therefore, it is discussed if microRNA expression could be used as biomarker for response to TKI treatment (Litwinska and Machalinski, 2017).

It has to be added that SNVs in the 3’ UTRs of microRNA target genes, as well as expression of alternate 3′ UTR lengths might tremendously affect microRNA binding resulting in tumor cell escape from therapy (Kasinski and Slack, 2011). This was shown e.g., for *ABCB1* and *ABCG2* in various cancer cell lines (To et al., 2008; Bruhn et al., 2016), as well as for three members of the ABCC family (Bruhn et al., 2020). Moreover, binding of let-7 was impaired by mutated *KRAS* 3’ UTR (Chin et al., 2008).

### Alternative Mechanisms of Resistance

The constitutive activation of the BCR-ABL1 fusion protein leads to pleiotropic stimulation of various signaling pathways involving JAK/STAT, MAP-kinases and PI3K/Akt signaling pathways. These result in increased cell proliferation, anti-apoptotic signaling, as well as altered cell motility and adhesion to stroma cells (Cilloni and Saglio, 2012). As the majority of these signaling pathways are oncogene addicted to BCR-ABL1 activity, treatment with BCR-ABL1 inhibitors is highly successful. However, these pathways can be captured by alternate stimuli, as shown e.g. for WNT/β-catenin signaling in leukemic stem cells or JAK2 activation by external stimuli (Braun et al., 2020), which makes the tumor cell at least partially autonomous from BCR-ABL1 potentially facilitating therapy failure or unsatisfactory response rates. Moreover, adaptions of the signaling pathways cannot only occur due to differential gene expression, but also due to mutations downstream of BCR-ABL1 or in alternative signaling pathways. These include re-activation of proliferative pathways, e.g. hedgehog or PI3K/Akt signaling, or activation of autophagy (comprehensively reviewed in (Minciacchi et al., 2021). The main difficulty with this is the detection of the responsible signaling pathways to find a suitable target (and drug) combination to circumvent resistance and trigger synthetic lethality, especially of leukemic stem cells (Cilloni and Saglio, 2012). As TKIs–at least to date–require a life-long therapy, they promote the development of mutations, clonal evolution and selection, which facilitates CML progression, but also TKI resistance and thereby adaption of the therapeutic strategy. Luckily, in cases of imatinib failure, a switch to newer generation TKIs according to the guidelines leads to good responses in most patients (Baccarani et al., 2013; Hochhaus et al., 2020).

### How to Analyze Genomics of Drug Resistance: In Vitro-Models

As drug resistant cell lines can hardly be established from primary material, these cell lines are utterly important to investigate drug responses. Although these tools are necessary to understand the biology and the mechanisms of drug resistance, some cancer cell lines potentially differ from the tumor they derived from and the transfer to the clinical situation might be limited (Sandberg and Ernberg, 2005; Ertel et al., 2006). Nevertheless, studies on drug efficacy using cell lines were successfully transferred to cancer patients, as shown e.g. for prediction of drug efficacy using gene expression data of cell lines by artificial intelligence and machine-learning (Borisov et al., 2018). In addition, cell lines have been used to develop treatment protocols, as shown for CML using K-562, but also NB4 cells for acute promyelocytic leukemia (Mirabelli et al., 2019). The application of drug resistant cell lines appears still to be the best model to analyze drug resistance (Rumjanek et al., 2013). These can either be generated by pulse treatment or continuous administration of increasing drug concentrations to a given cell line (McDermott et al., 2014). Regarding CML drug resistance models, the majority of studies have been performed on the K-562 cell line (e.g., Turrini et al., 2012; Kaehler et al., 2017), but other cell lines, e.g. LAMA-84 or KCL-22, have been tested as well. The major drawback with these cell lines is their origin in blast crisis of CML patients, which might not reflect the clinical situation of treatment of chronic phases, where initial therapy failure is observed. Therefore, the use of cell lines always implies future studies for the transfer of the observed resistance mechanisms to the clinical situation.

## Discussion

Targeted treatment of CML using specific tyrosine kinase inhibitors of the causal BCR-ABL1 fusion protein is tremendously successful. With this it was shown that targeting a single protein in the tumor cell can lead to therapeutic remission. Since CML cells are highly oncogene addicted to BCR-ABL1, inhibition of this protein and its downstream signaling pathways is sufficient to promote the demise of the tumor cells. This strategy was transferred to other tumors and is especially successful whenever the tumor cells have a high dependency on druggable kinases and has a rather simple complexity. Additional examples are HER2 inhibition in breast cancer using lapatinib and/or HER2-specific monoclonal antibodies trastuzumab and pertuzumab or BRAF mutated malignant melanoma using the tyrosine kinase inhibitors vemurafenib or dabrafenib (Swain et al., 2015; Jiao et al., 2018). While therapy using HER2-inhibition is genuinely successful in primary or advanced HER2-positive breast cancer, BRAF inhibition is often undertaken by downstream mutations leading to time-dependent relapses (Finn et al., 2012; Pernas and Tolaney, 2019). Therefore, BRAF inhibition is often combined with immune checkpoint inhibitors, i.e., ipilimumab, nivolumab or pembrolizumab, while drastically improved the outcome (Furue et al., 2018). CML still is one of the few neoplasms, in which a single agent can be used to successfully treat the disease, as for others, the combination of different agents often exceeds the response rates of a monotherapy and reduces the likelihood of drug resistance (Palmer and Sorger, 2017; Jardim et al., 2020). To this day, a variety of TKIs can be used for several tumors and are mainly administered in a co-treatment strategy (Jiao et al., 2018). Nevertheless, identification of the right (sub) population of tumors is often the key for successful therapy.

Regarding mechanisms of drug resistance, the findings in TKI resistant CML are likely to be transferrable to other drug-tumor combinations. This is the case as e.g. the majority of TKIs are metabolized by CYP3A4 and transported by ABC efflux transporters (Di Gion et al., 2011; Scheffler et al., 2011). Studying various combinations of anti-cancer drugs and tumor entities, it can be concluded that drug-drug interactions and pharmacogenetic variants might play a role in the development of drug resistance in other drug-tumor combinations. However, a predictive role for these variants at least for ABC transporters is not possible yet (Bruckmueller and Cascorbi, 2021).

As shown for BCR-ABL1, mutations in the binding domain of a respective kinase inhibitor or its overexpression/gene amplification have been observed in multiple tumors leading to drug resistance, as shown for acquired EGFR T790M mutations and c-MET receptor tyrosine kinase amplification promoting gefitinib resistance in lung cancer or KIT exon 14 or 17 and PDGFRA exon 14 mutations providing resistance against imatinib and reduced efficacy of sunitinib in GIST (Lynch et al., 2004; Gao et al., 2013; Kobayashi et al., 2013; Zhang et al., 2019). In addition, observations derived from CML regarding activation of alternative signaling pathways can also be observed in other tumor entities. This shows that processes of drug resistance observable in CML are highly similar to other drug-tumor combinations.

Overall, the genomics of impaired response against tyrosine kinase inhibitors observed in CML (Figure 1) might be observed during the treatment of other tumors using alternate TKIs as well. Mechanisms of resistance against TKIs often consist of a variety of layers, on mutations of the TKI target gene, in metabolic enzymes, drug transporters or in proteins of downstream or alternative signaling pathways. Adaption of the therapeutic regimen and development of new compounds overcoming these obstacles are necessary to further improve therapy response to TKIs.
